# Barriers to the utilization of the Sehaty mobile health application in Saudi Arabia: a cross-sectional survey of non-healthcare users

**DOI:** 10.3389/fmed.2025.1554078

**Published:** 2025-04-09

**Authors:** Haitham Alzghaibi

**Affiliations:** Department of Health Informatics, College of Applied Medical Sciences, Qassim University, Buraydah, Saudi Arabia

**Keywords:** mHealth, Sehaty app, survey, cross-sectional, barriers

## Abstract

**Introduction:**

Mobile health (mHealth) applications have the potential to enhance healthcare accessibility and management. However, several barriers continue to hinder their widespread adoption. In Saudi Arabia, the Sehaty app plays a critical role in national digital health efforts, yet little is known about the challenges faced by non-healthcare users.

**Methods:**

A cross-sectional survey was conducted among 403 non-healthcare users of the Sehaty app. Data were collected using a structured, validated questionnaire assessing ten categories of barriers: technical limitations, usability challenges, accessibility constraints, privacy and security concerns, communication and interaction difficulties, functionality restrictions, user satisfaction deficits, cost-related issues, time and productivity constraints, and inadequate support and training. Descriptive statistics, correlation analysis, and group comparisons using one-way ANOVA and t-tests were performed.

**Results:**

Technical issues such as frequent crashes and slow response times were reported as the most prominent barriers, significantly affecting user satisfaction. Usability challenges, including unintuitive navigation, were also widely reported. Privacy and security concerns—particularly regarding transparency and data protection—were major deterrents to app use. Accessibility constraints were more pronounced among older adults and those with disabilities, often compounded by limited support and training. Time inefficiencies and limited app functionality further reduced engagement. Cost-related barriers were minimal. Correlation analysis revealed strong associations among technical, usability, and functionality barriers, while demographic comparisons showed no significant differences across user groups.

**Discussion:**

The findings underscore the need for comprehensive improvements to enhance the Sehaty app’s usability, reliability, security, and accessibility. Addressing these challenges through technical optimisation, user-centred design, enhanced data protection, and targeted training can support broader adoption and align the app’s development with Saudi Arabia’s Vision 2030 digital health goals.

## Introduction

Mobile health (mHealth) applications have emerged as transformative tools in healthcare, offering patients improved access to medical information, monitoring tools, and communication with providers (1, 2). Despite their potential, the adoption and sustained use of mHealth apps face significant barriers from the perspective of patients. These challenges span technical, usability, financial, and psychological domains, limiting the effectiveness and accessibility of these technologies (3–5).

One of the most prominent barriers is the usability of mHealth apps. Many patients find these apps difficult to navigate due to complex user interfaces or insufficient design considerations for diverse user needs. Developing a user-friendly interface for mHealth apps is crucial, especially considering the diverse user base that includes elderly patients who may not be tech-savvy. They struggle with app usability due to limited technological literacy and physical challenges such as poor vision or dexterity issues (5, 6). Additionally, the time commitment required to learn and use these apps can discourage busy individuals or those with limited motivation (6).

A lack of digital literacy among patients is another critical issue. Many individuals are unaware of the existence or benefits of mHealth apps, while others lack the technical skills to use them effectively (5–7). This gap is particularly evident among older populations and those in underserved communities where access to technology and education is limited (7, 8). Without targeted efforts to improve digital literacy, these groups remain excluded from the potential benefits of mHealth applications. Privacy and security concerns are significant deterrents for many patients considering mHealth apps. Users often worry about unauthorized access to sensitive health data or misuse of their personal information (6). These concerns are heightened for apps dealing with stigmatized conditions such as mental health or HIV/AIDS, where breaches could lead to social isolation or discrimination (3, 6). Moreover, many mHealth apps lack clear privacy policies or robust security features, further eroding trust among users (3, 6).

The integration of mHealth apps into existing healthcare systems presents another barrier. Many apps operate in isolation without seamless connectivity to electronic health records (EHRs) or healthcare providers’ systems (6, 7). This lack of interoperability limits their utility for both patients and clinicians. For example, patients may need to manually input data into apps that do not sync automatically with other platforms, creating additional burdens and reducing engagement (9, 10). Cost is a recurring issue for patients when adopting mHealth apps. While some apps are free, others require subscription fees or in-app purchases that may not be affordable for all users (10, 11). Hidden costs within supposedly free apps can also deter continued usage. Furthermore, the need for reliable internet connectivity and modern mobile devices adds an indirect financial burden for users in low-income settings (5).

The regulatory environment surrounding mHealth apps remains fragmented and unclear in many regions. Patients often question who is accountable for their care when using these tools—whether it is the app developers or healthcare providers (3, 5). Ethical concerns also arise regarding informed consent and data ownership, as many users are unaware of how their data is collected, stored, or shared (3). Patient motivation plays a crucial role in mHealth app adoption. Many individuals lack the drive to engage with these tools consistently, especially if they perceive minimal immediate benefits (6). Time constraints further exacerbate this issue; busy schedules make it challenging for patients to dedicate time to learning new technologies or inputting data regularly into apps (6).

Reliable technology infrastructure is essential for effective mHealth app usage but is often lacking in remote or underserved areas. Many rural and remote areas lack the necessary infrastructure for high-speed internet, which is crucial for seamless video consultations (7). Poor internet connectivity, outdated devices, and compatibility issues with certain operating systems hinder access to these tools for many patients (3). Without addressing these infrastructural gaps, the reach of mHealth applications remains limited.

The Sehhaty app, which translates to “My Health” in English, is a unified digital health platform developed by the Saudi Ministry of Health (MoH) (12). Launched as part of Saudi Arabia’s digital health transformation, Sehhaty serves as a comprehensive healthcare management tool for citizens and residents in the Kingdom (12, 13). The Sehhaty app in Saudi Arabia exemplifies the potential of mobile health applications to transform healthcare access and delivery. Sehhaty is designed to improve the patient experience by providing a comprehensive array of features, such as booking medical appointments, accessing teleconsultations, and managing prescriptions. The app allows users to access medical records, receive vaccination updates, and track vital health metrics, including step counts and heart rate, thereby fostering a comprehensive approach to health management (12).

The app has gained significant popularity, with over 24 million users, representing approximately 68.5% of Saudi Arabia’s population (14). During the COVID-19 pandemic, Sehhaty played a crucial role in the country’s response, facilitating over 24 million testing appointments and the administration of more than 61 million vaccine doses (14). The Sehaty app has become a cornerstone of healthcare delivery in Saudi Arabia, providing essential services such as virtual health visits, follow-ups, and consultations. As a platform mandated for use by all Saudi citizens and visitors, it plays a critical role in ensuring access to healthcare in alignment with the Saudi Vision 2030 goals of digital transformation (13). Understanding the barriers to the utilization of the Sehaty app is crucial for enhancing its effectiveness and ensuring equitable access to healthcare services. By addressing technical, usability, and accessibility challenges, this study contributes to optimizing the app’s functionality, ultimately supporting its role as a vital tool in the healthcare system for millions of users across the Kingdom.

Understanding the barriers to the utilization of the Sehaty app is crucial for enhancing its effectiveness and ensuring equitable access to healthcare services. By addressing technical, usability, and accessibility challenges, this study contributes to optimizing the app’s functionality, ultimately supporting its role as a vital tool in the healthcare system for millions of users across the Kingdom. Despite the app’s widespread use, gaps remain in understanding the specific challenges encountered by non-healthcare users. Current research on mHealth adoption often focuses on healthcare professionals or patients with chronic conditions, with limited exploration of broader public perceptions. This study seeks to bridge this gap by providing a comprehensive analysis of the barriers faced by the general Saudi population, offering data-driven insights that can inform policy, improve app design, and enhance user engagement.

### Study aim and objectives

This study aims to explore and analyze the primary barriers to the adoption and utilization of the Sehaty mobile health application in Saudi Arabia. By identifying key challenges, the study seeks to develop evidence-based recommendations to enhance the app’s effectiveness, usability, and overall user experience.

### Objectives

- To systematically examine the ten key barriers to Sehaty app adoption and use: technical limitations, usability challenges, accessibility constraints, privacy and security concerns, communication and interaction difficulties, functionality restrictions, user satisfaction deficits, cost-related barriers, time and productivity constraints, and inadequate support and training.
- To assess the prevalence and severity of these barriers among non-healthcare users in Saudi Arabia.
- To explore potential correlations between different barriers, highlighting how specific challenges may influence or exacerbate others.
- To evaluate whether demographic factors such as age, gender, and education level influence perceptions of these barriers.
- To provide actionable insights and recommendations for improving the Sehaty app’s design, accessibility, security, and functionality, ensuring alignment with Saudi Arabia’s Vision 2030 digital healthcare transformation goals.

### What this study adds

- This research establishes a comprehensive framework for evaluating mobile health application challenges by systematically classifying ten critical barriers into 45 operationalized items. This structured approach provides a robust methodology for assessing mHealth usability and adoption barriers.
- The study underscores the interconnected nature of these barriers, revealing how technical challenges, usability issues, and accessibility constraints collectively impact user engagement and adoption. Addressing one barrier, such as system stability, may have cascading benefits on usability and time efficiency.
- It highlights significant accessibility challenges among older adults and marginalized populations, advocating for targeted design enhancements, including larger fonts, simplified navigation, and tailored support systems, to bridge the digital divide and promote inclusivity.
- Privacy and security concerns are examined in depth, with findings indicating that trust in data security and transparency is crucial for sustained engagement. The study calls for enhanced data protection policies and user education to mitigate concerns regarding privacy risks.
- By employing one-way ANOVA and t-tests, the study assesses group differences in barrier perceptions and finds no statistically significant variation across demographic groups. This suggests that the identified barriers are universally experienced, reinforcing the need for broad system-wide improvements rather than targeted interventions for specific subgroups.
- The study contributes region-specific insights into mHealth adoption in Saudi Arabia, providing empirical evidence to support health policymakers, app developers, and digital health strategists in refining mobile health solutions that align with national healthcare objectives under Vision 2030.

## Methods

### Study design

This study employed a cross-sectional research design to systematically assess barriers encountered by users of the Sehaty app in Saudi Arabia. A cross-sectional approach was selected to facilitate a temporal snapshot of the challenges experienced by app users, allowing for an evaluation of potential impediments to its utilization at a specific moment in time.

### Population

The target population for this study comprised non-healthcare users of the Sehaty app in Saudi Arabia. This demographic represents a diverse cohort of mobile health application users, including patients and general citizens who engage with the platform for healthcare services but lack formal medical or health-related training.

#### Inclusion criteria

- Saudi citizens who have accessed the Sehaty app at least once.
- Individuals aged 18 years or older.
- Non-healthcare professionals with no formal education or training in medical or health-related disciplines.
- Participants willing to provide informed consent and complete the questionnaire.

#### Exclusion criteria

- Healthcare professionals, including physicians, nurses, and allied health practitioners, whose professional background may influence their perceptions of the app’s usability.
- Individuals who have never used the Sehaty app.
- Respondents below the age of 18.
- Incomplete or inconsistent questionnaire responses.

### Sample size

A total of 403 valid responses were obtained and analyzed in this study. Given that the target population includes all Saudi citizens who have used the Sehaty app, determining an appropriate sample size necessitated statistical justification. Considering Saudi Arabia’s total population of over 35 million, a robust sample size was required to ensure generalizability.

Using Cochran’s formula for sample size determination:


$$n=\frac{Z^{2}\times P\times \left(1-P\right)}{E^{2}}$$

where:

- *n* represents the required sample size,
- *Z* is the z-score corresponding to a 95% confidence level (1.96),
- *P* is the estimated proportion of Sehaty app users in the population (assumed at 50% for maximum variability),
- *E* is the margin of error (set at 5%).

The calculation yielded a minimum required sample size of 385 respondents. The collected 403 responses surpassed this threshold, thereby enhancing the statistical reliability and robustness of the findings.

### Sampling technique

A convenience sampling approach was employed to recruit participants. This non-probability sampling technique was selected due to its practicality, cost-effectiveness, and efficiency in rapidly obtaining responses from accessible users. While convenience sampling does not ensure full representativeness of the broader population, it enabled the study to capture insights from a diverse range of Sehaty app users within a limited timeframe.

### Instrument for data collection

Data collection was conducted using a structured questionnaire designed to systematically assess barriers to Sehaty app utilization. The questionnaire comprised three distinct sections:

1. *Assurance letter*: This introductory section provided participants with essential study-related information, ensuring transparency, ethical compliance, and informed consent. The assurance letter detailed the following:

- Study description: A concise explanation of the study’s objectives, emphasizing its focus on identifying barriers to Sehaty app utilization among non-healthcare users.
- Significance of participation: Participants were informed of the potential impact of their contributions in shaping future improvements to mobile health applications.
- Estimated time commitment: Respondents were notified that completing the questionnaire would take approximately 10–15 min.
- Voluntary nature of participation: It was explicitly stated that participation was entirely voluntary and that individuals retained the right to withdraw at any stage without repercussions.
- Confidentiality and data handling: Participants were assured that their responses would remain strictly confidential, anonymized, and utilized exclusively for research purposes. Data would be securely stored and analyzed in aggregate form, ensuring the protection of respondents’ identities.

2. *Demographic section*: This section collected background information to contextualize the findings. The demographic variables included:

- Age
- Gender
- Education level
- Frequency of Sehaty app usage
- Digital literacy level

3. Likert scale items: This section contained 45 items distributed across ten key variables, identified in the literature as critical barriers to mobile health application adoption and usage. The variables included:

- Technical barriers
- Usability barriers
- Support and training
- Accessibility barriers
- Privacy and security barriers
- Communication and interaction barriers
- Functionality barriers
- User satisfaction barriers
- Cost and accessibility barriers
- Time and productivity barriers

Each barrier was operationalized through 4–5 items, with responses recorded using a five-point Likert scale (ranging from 1 = Strongly Disagree to 5 = Strongly Agree). To facilitate interpretation, mean scores for each barrier were calculated, with higher scores indicating a stronger perception of the given barrier.

The questionnaire was developed based on an extensive review of existing literature on mobile health application adoption, ensuring that all identified barriers were well-grounded in prior research. The instrument underwent pre-testing through a pilot study involving 12 participants drawn from the target population. This process assessed the clarity, readability, and comprehensibility of the questionnaire items, leading to minor refinements before full deployment. Expert validation was also conducted, where specialists in health informatics and mobile health technologies reviewed the questionnaire for content validity and alignment with the study objectives.

The final version of the questionnaire was administered in Arabic, the primary language of the target population, to enhance comprehension and response accuracy. A professional translation process was employed, including forward and backward translation, to ensure linguistic and conceptual equivalence with the original English version.

### Method of data collection

The utilization of online and social media platforms, such as WhatsApp, for data collection presents several advantages over traditional face-to-face methods. Digital platforms have been shown to yield higher response rates, with studies indicating a 16% increase in responses compared to in-person surveys, underscoring their effectiveness in reaching participants and facilitating timely data collection (15). Moreover, online data collection enables researchers to obtain large and demographically diverse datasets at a lower cost, with greater flexibility, making it particularly advantageous for studies requiring broad geographical coverage (16). Additionally, digital platforms streamline data management and allow for real-time engagement with participants, mitigating logistical challenges associated with conventional data collection approaches (17). These findings highlight the increasing significance of digital media in contemporary research, demonstrating its capacity to enhance data accessibility, efficiency, and scalability.

In this study, data were collected through an online survey distributed to users of the Sehaty app across Saudi Arabia over a four-month period, from May to August 2024. The survey link was disseminated via multiple digital channels, including social media platforms and online community forums, to maximize outreach and participant engagement. The adoption of an online survey methodology facilitated access to a geographically diverse sample, enabling individuals from various regions of Saudi Arabia to participate without the logistical constraints associated with face-to-face data collection. Furthermore, online participation allowed respondents to complete the survey at their convenience, thereby minimizing temporal and spatial barriers often encountered in traditional survey methods. This approach not only ensured broader representation but also enhanced the efficiency and reliability of the data collection process.

To enhance participation, two follow-up reminders were sent one at the midpoint of the data collection period and another two weeks before the survey closed. These reminders served to encourage non-respondents to participate and helped mitigate response bias.

The extended data collection period allowed sufficient time to capture responses from a diverse and geographically distributed sample, reflecting a range of demographic backgrounds and user experiences. Participants were provided with clear instructions on survey completion, and technical support was available for those encountering difficulties accessing or submitting responses. This approach ensured a high response rate and improved the reliability of the data collected.

### Reliability and validity

To evaluate the reliability of the questionnaire, internal consistency was assessed using Cronbach’s alpha. This statistical measure was applied to the overall questionnaire as well as the individual ten barrier-related variables. A Cronbach’s alpha value exceeding 0.7 was deemed acceptable, indicating a high degree of internal consistency and reliability.

The validity of the instrument was established through a pilot study conducted prior to full-scale data collection. A sample of 12 participants, drawn from the target population, was recruited to test the questionnaire. These individuals provided feedback regarding item clarity, comprehension, and relevance. Based on their responses, necessary refinements were made to enhance the instrument’s construct validity. Furthermore, expert validation was conducted by subject matter specialists in health informatics to ensure content relevance and alignment with the study’s objectives.

### Data analysis

Data analysis was conducted using SPSS v29, employing both descriptive and inferential statistical techniques. Descriptive statistics, including frequencies, percentages, and means, were calculated for each of the 45 items to summarize participants’ responses and provide insights into the prevalence and severity of perceived barriers to the utilization of mobile health applications. To assess the reliability of the questionnaire, Cronbach’s alpha was used to evaluate its internal consistency, both for the overall instrument and across the ten distinct variables representing different dimensions of barriers to the adoption of the Sehaty app.

Inferential statistical analyses were performed to examine potential differences in participants’ responses across various demographic groups. In addition, correlation analyses were conducted to explore the relationships between key variables, offering a deeper understanding of the interconnected nature of barriers to mobile health application usage.

To enhance data visualization, R software was utilized to illustrate the correlations among the ten primary variables (themes). These correlation analyses provided valuable insights into the interrelationships between different barriers, highlighting potential dependencies and areas requiring further investigation or intervention.

## Results

As seen in Table 1 the questionnaire’s reliability was evaluated using Cronbach’s alpha, demonstrating robust internal consistency among all variables and the overall instrument. The Cronbach’s alpha values for individual variables varied from 0.76 (Functionality Barriers) to 0.92 (Accessibility Barriers), demonstrating strong reliability for each item subset. The questionnaire exhibited exceptional reliability, evidenced by a Cronbach’s alpha of 0.95, affirming that the tool is highly dependable for assessing barriers to the use of the Sehaty app. The results indicate that the questionnaire is reliable and effectively captures participants’ perceptions across several aspects.

**Table 1:** Scale reliability using Cronbach’s alpha test.

Variables	Number of items	Cronbach’s Alpha
Technical barriers	5	0.85
Usability barriers	5	0.88
Accessibility barriers	4	0.92
Support and training barriers	5	0.79
Privacy and security barriers	4	0.89
Communication and interaction barriers	4	0.90
Functionality barriers	4	0.76
User satisfaction barriers	5	0.83
Cost and accessibility barriers	5	0.83
Time and productivity barriers	4	0.89
Entire questionnaire	45	0.95

**Figure 1:**
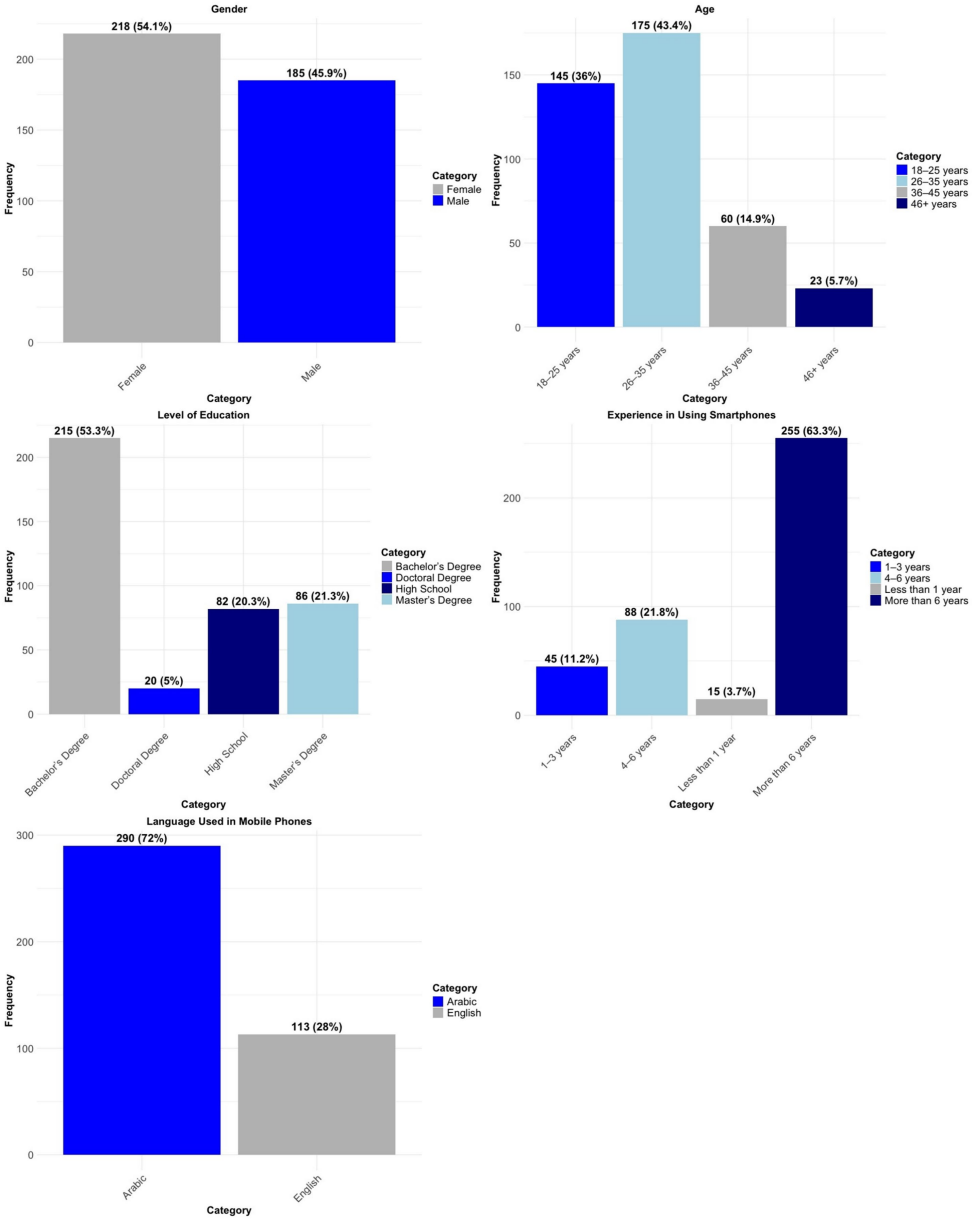
Demographic information of the participants.

Figure 1 summarizes the demographic features of the participants. The majority of participants were female, with the predominant age groups being 18–25 years and 26–35 years, constituting a substantial share of the sample. Concerning educational attainment, the majority of participants possessed a bachelor’s degree, followed by those with a Master’s degree, while fewer individuals reported holding high school diplomas or doctoral degrees. The majority of participants possessed over six years of smartphone usage experience, with a diminishing number in the groups of 4–6 years, 1–3 years, and under one year. The predominant language utilized on mobile phones was Arabic, as shown by the majority of participants, while English was employed by a lesser segment of the sample. The findings underscore the varied yet primarily younger, educated, and experienced composition of the participant group, exhibiting a pronounced preference for Arabic in mobile phone usage.

Table 2 presents the frequency and percentage of responses for each category, with values reported in the format of number of respondents (percentage). As shown in Table 2 the findings underscore various obstacles encountered by users in utilizing the application, particularly emphasizing technical and usability issues. The most significant technical barrier identified was frequent crashes or application instability, with a mean score of 4.02. Users experienced challenges in locating information within the application (Mean = 3.84), indicating navigation issues, whereas layout design garnered comparatively fewer criticisms (Mean = 2.09).

**Table 2:** Barriers distribution with percentages and mean.

Items	Strongly disagree	Disagree	Neutral	Agree	Strongly agree	Mean
Technical barriers						
The application often crashes or stops working while I am using it.	31 (7.69%)	30 (7.44%)	25 (6.2%)	132 (32.75%)	185 (45.91%)	4.02
The application is slow and unresponsive.	124 (31.0%)	160 (40.0%)	20 (5.0%)	57 (14.25%)	39 (9.75%)	2.32
The application often fails to connect to the internet.	163 (40.25%)	160 (39.51%)	25 (6.17%)	26 (6.42%)	31 (7.65%)	2.02
I experience frequent bugs or errors while using the application.	115 (28.54%)	157 (38.96%)	38 (9.43%)	52 (12.9%)	41 (10.17%)	2.37
The application does not work well on my device (phone, tablet, etc.).	136 (33.92%)	161 (40.15%)	26 (6.48%)	35 (8.73%)	43 (10.72%)	2.22
Usability barriers						
The application is difficult to use without help.	110 (27.5%)	158 (39.5%)	36 (9.0%)	56 (14.0%)	40 (10.0%)	2.4
I find it hard to navigate through the different features of the application.	131 (32.51%)	143 (35.48%)	42 (10.42%)	56 (13.9%)	31 (7.69%)	2.29
It takes too long to complete tasks, like booking appointments or viewing records, in the application.	135 (33.33%)	135 (33.33%)	29 (7.16%)	65 (16.05%)	41 (10.12%)	2.36
The application’s layout is confusing and not user-friendly.	152 (37.62%)	162 (40.1%)	28 (6.93%)	24 (5.94%)	38 (9.41%)	2.09
I often struggle to find the information I need within the application.	26 (6.48%)	63 (15.71%)	22 (5.49%)	129 (32.17%)	161 (40.15%)	3.84
Support and training barriers						
I did not receive any instructions or training on how to use the application.	137 (34.0%)	140 (34.74%)	53 (13.15%)	47 (11.66%)	26 (6.45%)	2.22
There is no help or support available when I have issues with the application.	143 (35.48%)	146 (36.23%)	46 (11.41%)	38 (9.43%)	30 (7.44%)	2.17
The instructions provided in the application are not clear or helpful.	127 (31.59%)	151 (37.56%)	55 (13.68%)	39 (9.7%)	30 (7.46%)	2.24
I do not know how to get assistance if the application does not work.	145 (36.16%)	146 (36.41%)	23 (5.74%)	51 (12.72%)	36 (8.98%)	2.22
Accessibility barriers						
The application is not accessible for people with disabilities (e.g., visual, hearing impairments).	124 (30.62%)	183 (45.19%)	31 (7.65%)	40 (9.88%)	27 (6.67%)	2.17
The font size and layout of the application make it difficult to read or use.	23 (5.72%)	52 (12.94%)	29 (7.21%)	144 (35.82%)	154 (38.31%)	3.88
The application is difficult to use for older people.	32 (7.94%)	57 (14.14%)	28 (6.95%)	134 (33.25%)	152 (37.72%)	3.79
I find it difficult to input information into the application.	120 (29.85%)	148 (36.82%)	35 (8.71%)	64 (15.92%)	35 (8.71%)	2.37
Privacy and security barriers						
I am concerned about the privacy of my personal and medical information in the application.	43 (10.59%)	23 (5.67%)	31 (7.64%)	142 (34.98%)	167 (41.13%)	3.9
I am worried that unauthorized people could access my data through the application.	114 (28.5%)	139 (34.75%)	49 (12.25%)	55 (13.75%)	43 (10.75%)	2.44
The application does not provide enough information about how my data is used.	33 (8.19%)	31 (7.69%)	42 (10.42%)	122 (30.27%)	175 (43.42%)	3.93
I do not trust the security of the application when it comes to protecting my medical records.	27 (6.73%)	42 (10.47%)	35 (8.73%)	125 (31.17%)	172 (42.89%)	3.93
Communication and interaction barriers						
The application makes it difficult to communicate with my healthcare providers.	140 (34.65%)	170 (42.08%)	35 (8.66%)	33 (8.17%)	26 (6.44%)	2.1
I do not receive timely responses from my healthcare provider through the application.	112 (27.79%)	152 (37.72%)	39 (9.68%)	60 (14.89%)	40 (9.93%)	2.41
I find it challenging to use the messaging or chat features in the application.	28 (7.02%)	32 (8.02%)	28 (7.02%)	133 (33.33%)	178 (44.61%)	4.01
The application does not allow me to interact effectively with my doctor or healthcare team.	125 (31.17%)	148 (36.91%)	55 (13.72%)	46 (11.47%)	27 (6.73%)	2.26
Functionality barriers						
The application lacks important features I need (e.g., scheduling appointments, viewing prescriptions).	125 (30.94%)	171 (42.33%)	34 (8.42%)	30 (7.43%)	44 (10.89%)	2.25
The application does not provide updated or accurate medical information.	129 (31.93%)	160 (39.6%)	31 (7.67%)	35 (8.66%)	49 (12.13%)	2.29
I find it hard to track my health or medical data using the application.	34 (8.44%)	31 (7.69%)	20 (4.96%)	126 (31.27%)	192 (47.64%)	4.02
The application does not integrate well with other health services I use.	127 (31.67%)	163 (40.65%)	42 (10.47%)	42 (10.47%)	27 (6.73%)	2.2
User satisfaction barriers						
I do not feel confident using the application.	21 (5.2%)	50 (12.38%)	24 (5.94%)	139 (34.41%)	170 (42.08%)	3.96
I do not find the application helpful for managing my health.	23 (5.71%)	41 (10.17%)	37 (9.18%)	111 (27.54%)	191 (47.39%)	4.01
The application is not improving my experience with healthcare.	133 (33.17%)	153 (38.15%)	48 (11.97%)	42 (10.47%)	25 (6.23%)	2.18
I would prefer to manage my healthcare without using this application.	38 (9.45%)	32 (7.96%)	35 (8.71%)	109 (27.11%)	188 (46.77%)	3.94
I would not recommend this application to others.	142 (35.32%)	138 (34.33%)	26 (6.47%)	56 (13.93%)	40 (9.95%)	2.29
Cost and accessibility barriers						
The application is too expensive or requires paid features to access important services.	160 (39.8%)	163 (40.55%)	24 (5.97%)	26 (6.47%)	29 (7.21%)	2.01
I cannot use the application because I do not have consistent access to the internet.	37 (9.16%)	23 (5.69%)	32 (7.92%)	129 (31.93%)	183 (45.3%)	3.99
My device (phone, tablet) is too old or incompatible with the application.	125 (30.86%)	150 (37.04%)	32 (7.9%)	67 (16.54%)	31 (7.65%)	2.33
The application requires too much data or memory on my device.	40 (9.93%)	36 (8.93%)	39 (9.68%)	125 (31.02%)	163 (40.45%)	3.83
Time and productivity barriers						
It takes too long to accomplish tasks in the application compared to other methods.	39 (9.65%)	30 (7.43%)	32 (7.92%)	140 (34.65%)	163 (40.35%)	3.89
The application adds unnecessary steps to managing my healthcare.	124 (30.62%)	157 (38.77%)	42 (10.37%)	48 (11.85%)	34 (8.4%)	2.29
Using the application does not save me time when managing my health.	32 (8.0%)	43 (10.75%)	35 (8.75%)	108 (27.0%)	182 (45.5%)	3.91
The application slows down my ability to book appointments or access services.	40 (9.93%)	34 (8.44%)	37 (9.18%)	132 (32.75%)	160 (39.7%)	3.84

Concerns regarding support, accessibility, and privacy were also significant. Users indicated a deficiency in instructions or training for application usage (Mean = 2.22), while accessibility issues for older users were identified as a notable barrier (Mean = 3.79). Privacy concerns, especially those related to data security and transparency, were significant, with the statement “The application does not provide enough information about how my data is used” receiving a mean score of 3.93. Barriers to communication and interaction adversely affected user experience, with messaging and chat features presenting notable challenges (Mean = 4.01).

Barriers to functionality and user satisfaction indicate areas requiring enhancement. Users indicated challenges in effectively tracking health data (Mean = 4.02) and expressed a lack of confidence in utilizing the application (Mean = 3.96). Cost concerns were minimal (Mean = 2.01), whereas consistent internet access was a significant issue (Mean = 3.99). The findings highlight the necessity for improved usability, enhanced support and training, better accessibility features, and increased transparency in data security to comprehensively address these barriers.

**Figure 2:**
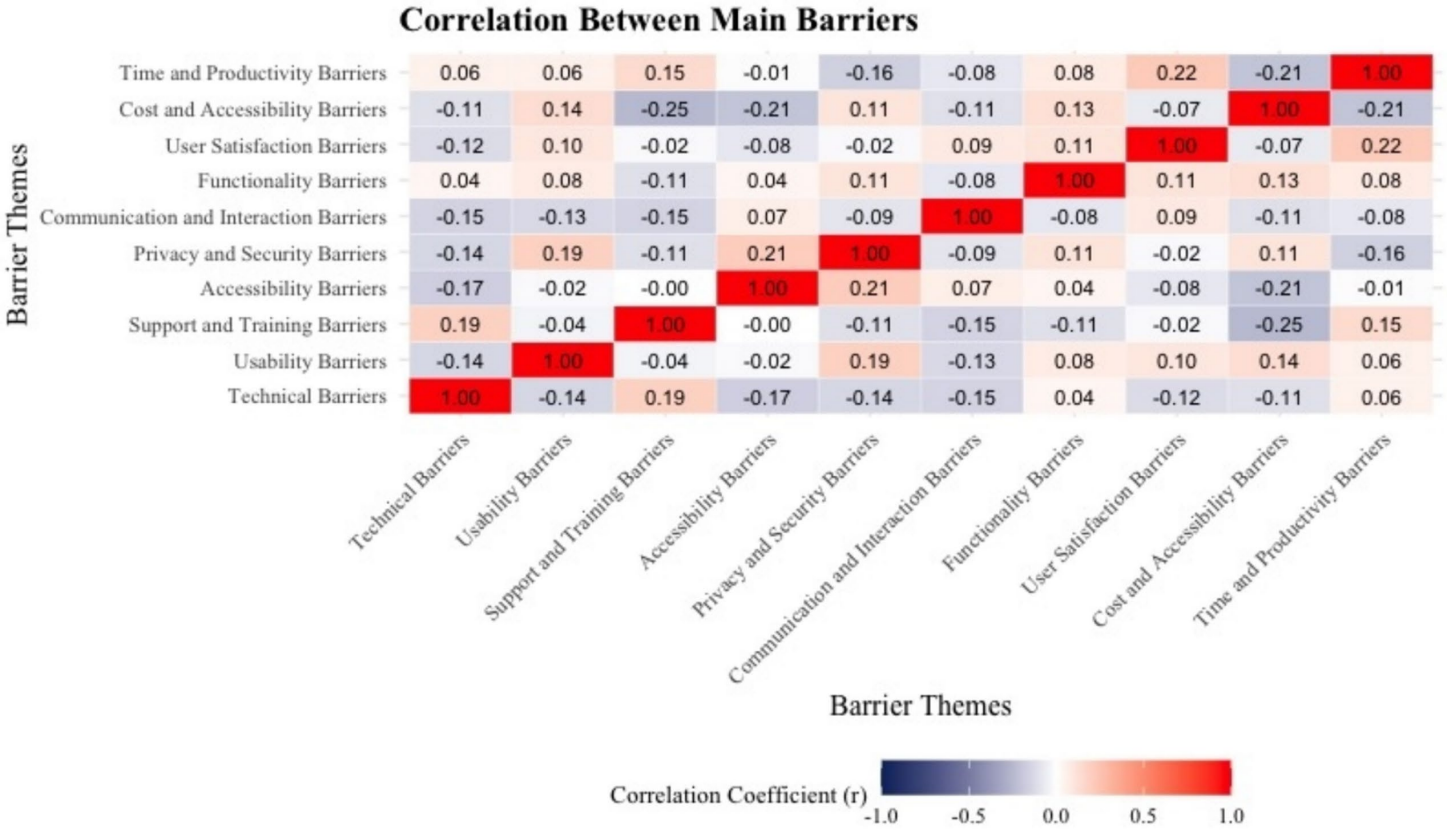
Correlation between main barriers.

The correlation analysis presented in Figure 2 reveals significant relationships among the primary barriers encountered by users. Notably, a strong correlation was identified between technical barriers and usability barriers (*r* = 0.80), suggesting that technical issues such as system crashes and instability have a direct and substantial impact on user experience and ease of navigation. Similarly, technical barriers and functionality barriers (*r* = 0.77) exhibited a strong association, indicating that performance-related inefficiencies hinder essential application features, thereby reducing overall usability. User satisfaction was found to be significantly correlated with privacy and security barriers (*r* = −0.44), highlighting the crucial role of data protection and transparency in fostering trust. This negative correlation suggests that heightened privacy and security concerns are associated with lower user satisfaction, reinforcing the necessity of robust data protection measures.

Moderate correlations were observed between accessibility barriers and support and training barriers (*r* = 0.45), as well as between time and productivity barriers and cost and accessibility barriers (*r* = 0.42). These findings indicate that accessibility challenges frequently stem from insufficient training and support, while inefficiencies in time management are often linked to broader accessibility constraints. Conversely, weak correlations were found between cost barriers and technical barriers (*r* = 0.06), suggesting that financial constraints and technological performance issues operate largely independently. The relatively low correlation between communication and interaction barriers and usability barriers (*r* = 0.25) further indicates that while usability issues may contribute to communication difficulties, they do not serve as the primary determinant.

These findings underscore the necessity of a comprehensive, multi-faceted approach to improving the Sehaty app, with a particular focus on technical reliability, usability, security, and accessibility to enhance user satisfaction and mitigate barriers effectively. Addressing technical instabilities is likely to yield broader benefits, improving usability, functionality, and user engagement with the application.

## Discussion

The findings of this study reveal significant barriers to the utilization of the Sehaty app among patients in Saudi Arabia, with technical and usability issues emerging as the most critical. Frequent app crashes, slow responsiveness, and navigation challenges were highlighted as primary obstacles, leading to dissatisfaction among users. These findings align closely with Giebel et al. (5) and Tea et al. (18), who identified technical instability as a leading deterrent to the adoption of digital health applications, negatively affecting user trust and engagement. Similarly, usability concerns, such as difficulties in locating essential features and the complexity of the user interface, were consistent with Khamaj and Ali (4), Zhou et al. (6), Tea et al. (18), and Ali (19), who emphasized the impact of poor design and navigation issues on mHealth app adoption. Addressing these technical and usability barriers is imperative for improving user satisfaction and ensuring the app’s success in supporting healthcare delivery.

Privacy and security concerns also emerged as major barriers in this study, as participants expressed apprehension about the safety and transparency of their personal and medical data. This aligns with findings by Kansiime et al. (3), Alfawzan et al. (20), Nurgalieva et al. (21), and Iwaya et al. (22), which reported that privacy-related concerns are a significant impediment to mHealth adoption, especially when users are unsure about data collection and usage policies. The Sehaty app must enhance transparency in data practices and implement robust security measures to mitigate these concerns and foster greater trust among users. Additionally, accessibility barriers were highlighted as significant challenges, particularly for older adults and individuals with disabilities. These findings are consistent with Byambasuren et al. (8) and Kao and Liebovitz (23), who noted that underserved populations, including the elderly, face challenges in adopting mHealth tools due to limited accessibility features and insufficient support. Improving accessibility through larger fonts, simplified navigation, and comprehensive tutorials could expand the app’s usability for diverse user groups.

Interestingly, cost was not identified as a significant barrier in this study, contrasting with findings from studies conducted in low-income settings where financial constraints hinder mHealth adoption (24, 25). This discrepancy may reflect the subsidized nature of the Sehaty app and its alignment with Saudi Arabia’s Vision 2030 initiative, which aims to enhance digital healthcare access. However, time-related barriers, such as inefficiencies in task completion and redundant workflows, were reported, mirroring the concerns raised by Abelson et al. (9), Zakerabasali et al. (11), Hengst et al. (26), and Patel and Shortliffe (27), who found that poorly designed mHealth apps often fail to optimize healthcare management tasks. These findings highlight the interconnected nature of barriers, where resolving one issue, such as technical instability, can positively influence others, including usability and user satisfaction. A holistic approach focusing on technical stability, accessibility, and user-centered design is essential to maximize the effectiveness and adoption of the Sehaty app, contributing to improved healthcare access and equity in Saudi Arabia.

Statistical analyses, including one-way ANOVA and independent t-tests, were conducted to examine potential differences in perceived barriers across gender, education level, and age groups. The results indicated no statistically significant differences among these demographic groups, suggesting that the challenges associated with the Sehaty app are consistent across diverse user segments. This finding implies that barriers to adoption and utilization are experienced uniformly, regardless of demographic variations, highlighting the need for broad, system-wide improvements rather than targeted interventions for specific user subgroups.

This research possesses multiple strengths. Initially, it offers a thorough evaluation of obstacles to mHealth application adoption from the patients’ viewpoint, concentrating on the prevalent Sehaty app in Saudi Arabia. The study provides a comprehensive picture of the obstacles users encounter by examining various elements, including technological, usability, privacy, accessibility, and temporal limitations. The incorporation of several demographic groups, including older persons and individuals with impairments, increases the study’s significance for marginalized communities. The utilization of a systematic questionnaire guarantees uniformity and comparability across participants, while the incorporation of statistical analysis, including correlation assessments, enhances the findings’ depth.

Nonetheless, this study has certain limitations. The reliance on self-reported data may introduce response bias, as participants’ perceptions and experiences may not fully align with objective assessments of the app’s performance. Additionally, individuals who do not use the Sehaty app may not have participated, leading to a potential omission of valuable feedback from those who have chosen not to adopt the platform. Their perspectives could provide critical insights into barriers to initial adoption, which are not captured in this study. Furthermore, the cross-sectional design restricts the ability to infer causal relationships between the identified barriers and the extent of app adoption. While the sample size is statistically sufficient, it may not fully represent all demographic groups in Saudi Arabia, particularly those in rural and underserved areas, where digital health accessibility and usage patterns may differ. This limitation affects the generalization of findings beyond the surveyed population. Moreover, the results are specific to the Sehaty app and may not be directly applicable to other mHealth platforms operating in different cultural, linguistic, or healthcare settings. Future research should address these constraints by incorporating longitudinal study designs, objective performance metrics, and comparative analyses across various mHealth applications to enhance the generalizability and depth of insights into mobile health adoption and utilization.

## Conclusion

The findings of this study highlight the necessity of a comprehensive, user-centered approach to enhancing the Sehaty mobile health application. Rather than addressing individual barriers in isolation, an integrated strategy that prioritizes technical stability, usability, privacy, and accessibility is imperative for fostering greater adoption and sustained user engagement. Enhancing data security measures and ensuring transparency in privacy policies will be pivotal in cultivating user trust and promoting wider utilization. Furthermore, the incorporation of inclusive design principles, such as larger font sizes, intuitive navigation, and tailored support for older adults and individuals with disabilities, is essential for mitigating digital accessibility challenges. While cost-related concerns were minimal, inefficiencies in time management and workflow integration underscore the need for optimizing system performance to improve overall efficiency and user experience. A holistic, evidence-based approach to refining the Sehaty app is required to align with Saudi Arabia’s Vision 2030 healthcare objectives and support the digital transformation of healthcare services. Future research should explore longitudinal trends in user engagement, objective performance metrics, and comparative analyses of mHealth platforms to further inform the development of sustainable, effective, and user-friendly digital health solutions.

## Data Availability

The raw data supporting the conclusions of this article will be made available by the authors, without undue reservation.
